# Partnership and relationship happiness in endometriosis related chronic pelvic pain: a multicenter case–control study

**DOI:** 10.3389/fpsyg.2024.1382067

**Published:** 2024-10-14

**Authors:** Samia el Hadad, Alexandra Sabrina Kohl Schwartz, Clarissa Gassner, Felix Haeberlin, Stephanie von Orelli, Patrick Imesch, Brigitte Leeners

**Affiliations:** ^1^Department of Reproductive Endocrinology, University Hospital Zurich, Zurich, Switzerland; ^2^Department of Reproductive Medicine, Cantonal Hospital Lucerne, Lucerne, Switzerland; ^3^Department of Reproductive Endocrinology, University Hospital Zurich, Zurich, Switzerland; ^4^Department of Obstetrics and Gynecology, University of Bern, Bern, Switzerland; ^5^Department of Gynecology and Obstetrics, Cantonal Hospital St. Gallen, St. Gallen, Switzerland; ^6^Department of Gynecology and Obstetrics, Triemli Hospital Zurich, Zurich, Switzerland; ^7^Department of Gynecology, University Hospital Zurich, Zurich, Switzerland; ^8^Faculty of Medicine, University Hospital Zurich, Zurich, Switzerland

**Keywords:** endometriosis, chronic pain, dyspareunia, dysmenorrhea, partnership, separation

## Abstract

**Introduction:**

Partnership is an important resource in dealing with endometriosis related chronic pain. Hence, our objective was to assess partnership in the context of endometriosis and its symptoms, considering the perspectives of both individuals involved.

**Methods:**

The study was designed as a multi-center matched case–control study in Switzerland, Germany and Austria. Altogether 381 women with surgically/histologically confirmed endometriosis and 381 control women, 250 male partners of endometriosis-affected women and 229 of control women were evaluated. Partnership quality, partnership happiness, separation thoughts, and areas of conflict were evaluated through the Partnership Questionnaire and a validated list of conflict areas.

**Results:**

Quality of partnership was rated as high by 60.1% of the women with endometriosis and 66.7% of the control women, as well as by 45.8 and 50.2% of their respective partners. Women with endometriosis mentioned separation thoughts, mostly related to sexual satisfaction, more often (34.9%/28.3%) and experienced more partnership-related conflicts than control women. Chronic pain, dyspareunia, dissatisfaction with sexuality, fatigue, and infertility were significantly associated with partnership conflicts. Fatigue and infertility but not pain experiences were related to lower partnership quality. Male partners in both groups reported separation thoughts equally often. In men, a high intensity of pain experienced by their partner was associated with reduced partnership happiness.

**Conclusion:**

Given the significance of partnership in dealing with chronic diseases and the connection between symptoms of endometriosis and a decrease in partnership quality, it is essential to incorporate strategies that alleviate the negative impacts on relationships for both partners into medical support.

**Clinical trial registration:**

identifier NCT 02511626.

## Introduction

Endometriosis affects 2 to 10% of women (Eisenberg et al., 2018; Eskenazi and Warner, 1997; Kennedy et al., 2005), with a prevalence of up to 50% in infertile women (Meuleman et al., 2009). Common symptoms include chronic pelvic pain, dyspareunia, dysmenorrhea, fatigue, and infertility (Kennedy et al., 2005; De Graaff et al., 2013; Ramin-Wright et al., 2018). These symptoms, along with the side effects of medical and surgical treatments, can significantly diminish women’s quality of life (Kennedy et al., 2005; Culley et al., 2013a; Denny, 2004; Kohl Schwartz et al., 2017; Kohl Schwartz et al., 2019; Leeners et al., 2018; Sperschneider et al., 2019). Despite the availability and clear guideline to define treatment strategies that help alleviate symptoms (Becker et al., 2022), as well as more and more refined surgical strategies to reduce post-operative pain development (Buzzaccarini et al., 2022) complete relief is often only partially achieved, and recurrence rates have been reported to reach up to 50% (Leuenberger et al., 2022; Nirgianakis et al., 2020; Seracchioli et al., 2010; Wacharachawana et al., 2021). As a result, many women must contend with persistent symptoms, particularly chronic pelvic pain (Leuenberger et al., 2022).

Chronic diseases and their symptoms for example pain can pose challenges to partnerships (Culley et al., 2017; Hämmerli et al., 2018). Previous studies have highlighted the adverse impact of endometriosis-related dyspareunia on sexual activity (Hämmerli et al., 2018; Bernays et al., 2020; Butt and Chesla, 2007; Fourquet et al., 2011; Fourquet et al., 2010), as well as the effects of endometriosis-related infertility and the substantial disruptions of social and leisure activities caused by endometriosis-related pain (Culley et al., 2017; Ameratunga et al., 2017; De Graaff et al., 2016). Notably, disease symptoms, especially consequences of pelvic pain have been linked to contributing factors in relationship separations (Denny, 2004; Hämmerli et al., 2018; Fagervold et al., 2009), and such separations have been identified as having a negative influence on the development of endometriosis (Buhmann et al., 2017; Müller et al., 2001). However, male partners play a crucial role as a significant source of support for women dealing with chronic diseases (De Graaff et al., 2013; Culley et al., 2013a; Denny, 2004) and reactions from close family and friends have been reported to significantly influence health-related quality of life in women diagnosed with endometriosis (Grundström et al., 2023).

Today medical health care professionals have become increasingly aware that adequate health care includes not only medical or surgical therapies, but also support to patients and their families in adjusting their daily lives to limitations and impairment brought on by chronic diseases. Although partnership represents a valuable resource for disease management, quality of life and overall health, few studies have investigated the association between the chronic gynecological pain disease endometriosis, endometriosis-related symptoms, and partnership quality (Culley et al., 2013a; Bernays et al., 2020; Butt and Chesla, 2007; Hudson et al., 2016; Schick et al., 2022; Rosen, 2001). Available studies focused on sexual behaviors only, did in most cases not include both partners, did not use validated questionnaires, and included only small sample sizes (Culley et al., 2013a; Butt and Chesla, 2007; Hudson et al., 2016).

Therefore, the objective of the present study was (i) to evaluate the association between endometriosis as a chronic gynecological pain disorder and partnership quality, areas of partnership conflicts, and separation thoughts; and (ii) to explore the role of specific endometriosis-related symptoms, especially endometriosis related pain in partnership quality. Our motivation was to identify areas for an optimization of medical care and support for women with endometriosis and their partners.

## Materials and methods

### Study design

Within a multi-center case–control study, questions in internationally validated questionnaires investigating different aspects of quality of life and risk factors for endometriosis were answered by women diagnosed with endometriosis and matched control women (Ramin-Wright et al., 2018; Kohl Schwartz et al., 2017; Hämmerli et al., 2018; Liebermann et al., 2018; Lukas et al., 2018). Questions on socio-demographic conditions, on sexuality, and on partnership were also answered by male partners.

The study was approved by the ethical committee of the Canton of Zurich, Switzerland (reference number: StV-Nr. 05/2008) as well as by the ethic boards of the participating hospitals. All study participants provided written informed consent for study participation. The study was conducted in agreement with the guidelines of the World Medical Association Declaration of Helsinki. The Strengthening the Reporting of Observational Studies in Epidemiology (STROBE) criteria were used to draft the manuscript (Elm et al., 2007). The trial registration number is NCT 02511626.

### Study group

Study participants had to be at least 18 years old, fluent in German, and able to understand and fill out the questionnaire. Only women with a histologically or surgically confirmed diagnosis of endometriosis, irrespective of their current disease symptoms were included in the case group. Women in the control group had to be either free of endometriosis-related symptoms or endometriosis had to be surgically excluded. In both groups other chronic pain symptoms (for example chronic headache or lower back pain) had to be reported, but were no exclusion criterion, while women with a current pregnancy were excluded. To have a more homogenous sample only couples in a current relationship providing answers to at least 90% of the questions were considered for evaluation. Women diagnosed with endometriosis and control woman were matched for age (±3 years).

### Recruitment

Women in Germany, Austria, and Switzerland were invited to participate in the study when presenting for gynecological consultations in hospitals or doctor’s offices or during hospital stays for benign gynecological disorders. To cover a broad range of women diagnosed with endometriosis and control women, study participants for both groups were recruited in university hospitals, district hospitals and private offices. A small number of the participants were recruited through endometriosis self-help groups. Only women without relevant physical and mental health issues other than the chronic pain conditions described above were included as control women. Consecutive referrals were evaluated over a 5-year study period. After verification of inclusion and exclusion criteria, information on the study and its aims was provided. Following written informed consent, each participant received the questionnaire. All women were asked to hand a separate questionnaire to their partners, which had to be completed. To support anonymity a second return envelope was provided with the male questionnaire to allow individual return. Men and women were asked to enter the first letter of their own and their partners name, first name and date of birth in the questionnaire, so that couples could be identified. A total of 743 women with a diagnosis of endometriosis and 1,322 control women were invited to participate in the study. We received 574 (77.3% of invitations) questionnaires from women with a diagnosis of endometriosis and 581 (43.9%) from control women. Of the initial 1,155 female study participants, 81.9% (*n* = 467) of the women diagnosed with endometriosis and 78.2% (*n* = 458) of the control women were in a current relationship (*p* = 0.198), with 67.0% (313) of partners of women with endometriosis and 60.9% (279) of partners from control women returning a completed questionnaire. After matching 762 women, i.e., 381 matched pairs, with 250 partners of women with endometriosis and 229 partners of control women were available for the present analysis. With the inclusion of 381 women diagnosed with endometriosis and 381 control women the study sample was slightly above the required 380 needed to detect a 5% difference with a power of 80%, an *α*-error of 0.05, and a *β*-error of 0.2.

### Partnership evaluation

Participants were asked about partnership status, as estimated by themselves and the duration of the current partnership (no current partnership, <1 year, 1–3 years, 3–7 years, 7–15 years, >15 years).

The quality of the partnership was investigated via the Partnership Questionnaire (Partnerschaftsfragebogen, PFB). The PFB is a well-established questionnaire used for patient care and research (Hahlweg, 2016; Richter et al., 2014). Reliability is good to very good for the total and for the subscores concerning argumentative behavior, tenderness, and togetherness/communication. Cronbach’s alpha for the total score is *α* = 0.94, and *α* = 0.90, *α* = 0.92, and α = 0.88 for the subscores. The retest reliability is between r_tt_ = 0.69 and r_tt_ = 0.85 for a time interval of 6 months. The PFB has very good discriminative and prognostic validity. It consists of 30 items separated into groups of 10 for each subscore. The frequency of observed behaviors of the partner is rated on a five-point scale: 0 (never/very rare); 1 (rare); 2 (often); 3 (very often); and 5 (irrelevant). One item addresses global happiness (5 answers from “very happy” to “very unhappy”). A total value <52 implies an unsatisfying partnership, a value between 52 and 67 an average-quality partnership, and a value >67 a high-quality partnership (Hahlweg, 2016; Richter et al., 2014).

The Problem List (PL) is a well-established questionnaire used to identify conflict areas in a partnership and possible solutions, as well as to assess the therapeutic effect of couple therapy in clinical and scientific settings (Richter et al., 2014). The PL consists of 23 items. Participants can choose among the following answers: 0 (no conflicts); 1 (conflicts, but resolved successfully); 2 (conflicts, unresolved, frequent disputes); 3 (conflicts, but no conversations about them); and 4 (no answer). Only the answers of categories 2 and 3 are considered to be conflicts for the total score. High scores indicate a low quality of partnership with many conflict areas and vice versa. The Cronbach’s alpha of the PL is 0.82 and the re-test reliability is r_tt_ = 57 (Hahlweg, 2016; Richter et al., 2014).

In addition, participants were asked to indicate whether endometriosis had caused a burden on their partnership, whether they ever thought about separation, and whether endometriosis-related factors played a major role in these reflections.

Male partners of women with endometriosis were asked 9 specific questions about endometriosis, including subjective experiences related to the disease (e.g., if it was difficult for them to see their partners suffering or if they were concerned about their partner’s health). Other questions addressed whether they feared never having a child or if they were angry about a significant impact of endometriosis on their lives. These questions were answered on a 4-point scale (1 = never/very rarely to 4 = very often; 5 = no answer). These questions were initially developed with the clinical experiences of the research team, i.e., health care professionals providing medical and/or psychosocial support for women and couples facing endometriosis and afterwards discussed and refined with the leading board of the self-help group.

### Covariates

The number of children, duration of the relationship, sexual satisfaction, and endometriosis-related symptoms (i.e., chronic pain, intensity and frequency of pain, dyspareunia, dysmenorrhea, fatigue and infertility) were investigated for their association with partnership quality, relationship happiness, and areas of conflict in the partnership.

### Statistics

Statistical evaluation was performed with SPSS Statistics, version 25. Categorial variables were compared with Pearson Chi^2^ test and mean values of continuous variables were compared with student t-test. A *p* value <0.05 was considered statistically significant. To analyze the impact of endometriosis on the partnership and the confounding effects of different covariates such as the number of children, duration of the relationship, sexual satisfaction, and endometriosis-related symptoms, we performed multivariable logistic regression analysis. Associations were quantified as adjusted odds ratios.

## Results

Tables 1, 2 provide an overview of the socio-demographic data and summarize the characteristics of endometriosis and disease symptoms in women with endometriosis and in control women. Most of the study participants reported long-term relationships. The number of children was significantly higher in control women. Problems with achieving pregnancy and fertility treatments were reported more often in women diagnosed with endometriosis than in control women. One fifth (21.5%) of the women with endometriosis and 47.5% of the controls had children already.

**Table 1:** Socio-demographic data and different symptoms of women with endometriosis and control women.

		Endometriosis (*n* = 381)	Control women (*n* = 381)	*p*-values
Age	Mean years (± SD)	37.69 (±7.34)	37.80 (±7.51)	0.849^a^
Nationality	Swiss	166 (43.7%)	200 (52.6%)	0.000^b^
	German	175 (46.1%)	106 (27.9%)	
	Austrian	6 (1.6%)	11 (2.9%)	
	Others	33 (8.7%)	63 (16.6%)	
BMI	Mean BMI (± SD)	22,82 (4.31)	23,15 (3.97)	0.272^a^
Duration of relationship	< 1 year	25 (6.6%)	26 (7.0%)	0.132^b^
	1–3 years	37 (9.8%)	49 (13.1%)	
	3–7 years	99 (26.3%)	77 (20.6%)	
	7–15 years	127 (33.7%)	113 (30.3%)	
	> 15 years	89 (23.6%)	108 (29.0%)	
Level of education^c^	No/low level of education	51 (14.8%)	48 (13.2%)	0.438^b^
	High level education/ Apprenticeship	170 (49.3%)	169 (46.3%)	
	University degree	124 (35.9%)	148 (40.5%)	
Monthly income	No income	40 (11.1%)	63 (17.0%)	0.071^b^
	< CHF 3000 /< € 2,700	89 (24.7%)	104 (28.1%)	
	CHF 3001–6,000/ € 2,701–5,400	185 (51.4%)	144 (38.9%)	
	> CHF 6000 CHF/> € 5,400	46 (12.8%)	59 (15.9%)	
Children	0	264 (69.3%)	168 (44.1%)	0.000^b^
	1	52 (13.6%)	65 (17.1%)	
	2	48 (12.6%)	106 (27.8%)	
	>2	17 (4.5%)	42 (11.0%)	
Infertility	yes	174 (45.7%)	66 (17.3%)	0.000^b^
Chronic pain *n* = 378 E, *n* = 374 C		224 (59.3%)	45 (12.0%)	0.000^b^
Dysmenorrhea *n* = 369 E, *n* = 361 C		246 (66.7%)	102 (28.3%)	0.000^b^
Dyspareunia *n* = 370 E, *n* = 377 C		240 (64.9%)	109 (28.9%)	0.000^b^
Fatigue *n* = 375 E, *n* = 372 C		293 (78.1%)	204 (54.8%)	0.000^b^

a Student *t*-test.

b Pearson χ^2^-test.

c Level of education assessed with the questionnaire: No/low level = No graduation or secondary school certificate; High level/Apprenticeship = High school graduation, Vocational baccalaureate diploma or apprenticeship; University degree = University degree.

E, Endometriosis women; C, Control women.

**Table 2:** Characteristics of endometriosis.

Characteristic	Category	*n* (%)
Duration since diagnosis of endometriosis (*n* = 346)	< 1 year	104 (30.1%)
	1–5 years	150 (43.3%)
	5–10 years	60 (17.3%)
	> 10 years	32 (9.2%)
rASRM-stage (*n* = 378)	I	66 (17.5%)
	II	89 (23.5%)
	III	109 (28.8%)
	IV	114 (30.2%)
Number of surgical interventions (*n* = 348)	1	190 (54.6%)
	2–3	137 (39.4%)
	4–6	19 (5.6%)
	7–10	2 (0.6%)
Chronic pain because of endometriosis (*n* = 224)	Yes	196 (86.6%)
Average pain intensity in the last month (*n* = 216)	0–3	70 (32.4%)
	4–6	93 (43.1%)
	7–10	53 (24.5%)
Frequency of endometriosis- related pain (*n* = 219)	Daily	74 (33.8%)
	Several times per week	62 (28.3%)
	Several times per month	55 (25.1%)
	Several times per year	28 (12.8%)

rASRM, revised American Society of Reproductive Medicine.

Quality of partnership was rated as high by 60.1% of the women with endometriosis and 66.7% of the control women (*p* = 0.150), as well as by 45.8% of partners of women afflicted with endometriosis and 50.2% of control partners (*p =* 0.595).

Results concerning thoughts about separation, the quality of partnership, and different areas of conflict— in women diagnosed with endometriosis, in control women, and in their male partners—are presented in Table 3. Tables 4a–d and Supplementary Tables 4e–h present multivariable regression analysis to evaluate associations between endometriosis and the quality of the partnership, controlling for different confounding effects. Time since first diagnosis was significantly shorter in women having thoughts about a separation from their current partner than in women who did not experience such thoughts (time since diagnosis <5 years: 92.2% vs. 77.6%; and > 5 years: 7.8% vs. 22.4%; *p* = 0.001). In contrast, there was no difference in rARSM stage between endometriosis-affected women with separation thoughts and those without such reflections (stage I: 14.4% vs. 19.6%; stage II: 22.7% vs. 24.3%; stage III: 31.1% vs. 29.4% and stage IV: 31.8% vs. 29.4%; *p* = 0.558).

**Table 3:** Quality of partnership as measured by the partnership questionnaire (PFB) and the Problem List (PL).

		Endo *n* = 381	Partners Endo *n* = 250	Endo/Partner *p* values	Controls *n* = 381	Partner Controls *n* = 229	Controls/Partner *p* values	Endo/Controls *p* values	Partner Endo/Partner Controls *p* values
Thoughts about separation^a^		133 (34.9%)	51 (20.5%)	0.000*	105 (28.3%)	57 (25.2%)	0.234	0.028*	0.131
**Total score PFB** ^a^	< 52	51 (13.4%)	44 (17.7%)	0.002*	36 (9.4%)	39 (17.2%)	0.000*	0.150	0.595
	52–66	101 (26.5%)	91 (36.5%)		91 (23.9%)	74 (32.6%)			
	> 67	229 (60.1%)	114 (45.8%)		254 (66.7%)	114 (50.2%)			
Argumentative behavior^a^	Low (0–7)	6 (1.6%)	6 (2.4%)	0.046*	2 (0.5%)	1 (0.4%)	0.666	0.331	0.137
	Intermediate (8–13)	8 (2.1%)	14 (5.6%)		10 (2.6%)	9 (3.9%)			
	High (14–30)	367 (96.3%)	230 (92.0%)		366 (96.1%)	218 (95.6%)			
Tenderness^a^	Low (0–12)	34 (9.0%)	41 (16.5%)	0.000*	18 (4.7%)	34 (14.8%)	0.000*	0.074	0.817
	Intermediate (13–18)	66 (17.5%)	62 (25.0%)		70 (18.6%)	55 (24.0%)			
	High (19–30)	278 (73.5%)	145 (58.5%)		288 (76.6%)	140 (61.1%)			
Togetherness/Communication^a^	Low (0–13)	26 (6.8%)	3 (1.2%)	0.004*	25 (6.6%)	7 (3.1%)	0.077	0.480	0.361
	Intermediate (14–19)	89 (23.4%)	62 (24.8%)		75 (19.8%)	57 (24.9%)			
	High (20–30)	266 (69.8%)	185 (74.0%)		278 (73.5%)	165 (72.1%)			
Happiness with the relationship^a^	Happy	340 (90.7%)	230 (92.4%)	0.278	345 (92.0%)	215 (95.1%)	0.181	0.604	0.547
	Unhappy	35 (9.3%)	19(7.6%)		30 (8.0%)	11 (4.9%)			
**Total score PL** ^b^	Mean ± SD	2.00 ± 3.36	1.69 ± 3.07	0.247	1.46 ± 2.91	1.18 ± 2.16	0.214	0.018*	0.140
	Conflicts with Monthly income	23 (6.0%)	14 (5.6%)	0.864	17 (4.5%)	6 (2.6%)	0.281	0.417	0.115
Job		29 (7.6%)	19 (7.6%)	1.000	33 (8.7%)	13 (5.7%)	0.206	0.691	0.466
Household		40 (10.5%)	35 (14.0%)	0.209	29 (7.6%)	18 (7.9%)	1.000	0.207	0.041*
Raising children		24 (6.3%)	13 (5.2%)	0.608	20 (5.2%)	15 (6.6%)	0.590	0.642	0.563
Leisure time		24 (6.3%)	8 (3.2%)	0.096	30 (7.9%)	15 (6.6%)	0.632	0.481	0.092
Friends		22 (5.8%)	17 (6.8%)	0.615	15 (3.9%)	8 (3.5%)	0.830	0.312	0.149
Temperament of partner		56 (14.7%)	25 (10.0%)	0.090	37 (9.7%)	13 (5.7%)	0.093	0.046*	0.092
Care		42 (11.0%)	27 (10.8%)	1.000	34 (8.9%)	20 (8.7%)	1.000	0.398	0.539
Attractiveness		21 (5.5%)	10 (4.0%)	0.454	10 (2.6%)	6 (2.6%)	1.000	0.065	0.454
Trust		35 (9.2%)	15 (6.0%)	0.175	19 (5.0%)	8 (3.5%)	0.424	0.033*	0.285
Jealousness		38 (10.0%)	18 (7.2%)	0.255	21 (5.5%)	11 (4.8%)	0.852	0.029*	0.339
Personal freedom		27 (7.1%)	12 (4.8%)	0.311	17 (4.5%)	8 (3.5%)	0.675	0.162	0.503
Sexuality		73 (19.2%)	48 (19.2%)	1.000	51 (13.4%)	31 (13.5%)	1.000	0.039*	0.109
Extramarital relationships		14 (3.7%)	4 (1.6%)	0.148	12 (3.1%)	7 (3.1%)	1.000	0.842	0.366
Relatives		42 (11.0%)	34 (13.6%)	0.382	39 (10.2%)	20 (8.7%)	0.575	0.841	0.112
Personal habits		52 (13.6%)	28 (11.2%)	0.394	41 (10.8%)	17 (7.4%)	0.200	0.268	0.163
Communication		35 (9.2%)	18 (7.2%)	0.463	32 (8.4%)	16 (7.0%)	0.642	0.798	1.000
Plans about having children		34 (8.9%)	16 (6.4%)	0.293	11 (2.9%)	5 (2.2%)	0.795	0.001*	0.026*
Missing acceptance/support		35 (9.2%)	14 (5.6%)	0.128	27 (7.1%)	7 (3.1%)	0.044*	0.354	0.189
Claims		32 (8.4%)	22 (8.8%)	0.885	24 (6.3%)	11 (4.8%)	0.478	0.331	0.104
Diseases		29 (7.6%)	11 (4.4%)	0.132	10 (2.6%)	4 (1.7%)	0.585	0.003*	0.118
Alcohol/Medicine/Drugs		27 (7.1%)	12 (4.8%)	0.311	13 (3.4%)	8 (3.5%)	1.000	0.034*	0.503
Assault		5 (1.3%)	0 (0.0%)	0.163	3 (0.8%)	1 (0.4%)	1.000	0.752	0.478

a Pearson χ^2^-test.

b Student *t*-test.

* Statistically significant (*p* < 0.05).

PFB, partnership questionnaire; PL, problem list.

**Table 4a:** Association between endometriosis as well as endometriosis related symptoms and thoughts about separation in women and in their male partners.

		Women			Partners		
		OR	95% CI	*p* value	OR	95% CI	*p* value
Endometriosis	Yes	1.202	[0.801; 1.776]	0.385	0.439**	[0.235; 0.818]	0.010
Number of children Ref: 0	> 2	0.574	[0.276; 1.193]	0.137	0.694	[0.282; 1.705]	0.425
	2	0.895	[0.556; 1.442]	0.649	0.804	[0.424; 1.527]	0.506
	1	0.748	[0.455; 1.227]	0.250	0.510	[0.243; 1.070]	0.075
Duration of relationship Ref: < 3 years	> 3 years	0.634	[0.369; 1.015]	0.058	1.224	[0.647; 2.317]	0.534
Happiness with sexuality	Unhappy	2.212**	[1.307; 3.743]	0.003	2.119**	[1.025; 4.377]	0.043
Chronic pain	Yes	0.858	[0.465; 1.582]	0.623	1.361	[0.527; 3.240]	0.486
Intensity of pain Ref: 0–3	7–10	0.969	[0.483; 1.986]	0.992	1.688	[0.597; 4.773]	0.324
	4–6	0.871	[0.474; 1.600]	0.656	2.542**	[1.104;5.853]	0.028
Frequency of pain Ref: Yearly/Monthly	Daily	1.047	[0.552; 1.985]	0.888	0.652	[0.264; 1.611]	0.354
	Weekly	1.330	[0.670; 2.643]	0.415	0.961	[0.394; 2.344]	0.930
Dyspareunia	Yes	1.566**	[1.070; 2.292]	0.021	1.260	[0.735; 2.160]	0.400
Dysmenorrhea	Yes	0.770	[0.523; 1.136]	0.188	0.766	[0.440; 1.333]	0.345
Fatigue	Yes	1.362	[0.921; 2.013]	0.121	1.196	[0.704; 2.033]	0.508
Infertility	Yes	1.089	[0.740; 1.602]	0.664	0.581	[0.315; 1.069]	0.081

Dependent variable: thoughts about separation: 0: no, 1: yes.

** Statistically significant (*p* < 0.05).

OR, Odds ratio; CI, Confidence interval.

**Table 4b:** Association between endometriosis as well as endometriosis related symptoms and the total score of the partnership questionnaire (PFB) in women and in their partners.

		Women			Partners		
		OR	95% CI	*p* value	OR	95% CI	*p* value
Endometriosis	Yes	0.876	[0.587; 1.306]	0.516	0.890	[0.551; 1.437]	0.635
Number of children Ref: 0	> 2	0.417**	[0.227; 0.765]	0.005	0.434**	[0.198; 0.949]	0.036
	2	0.456**	[0.293; 0.709]	< 0.001	0.500**	[0.290; 0.864]	0.013
	1	0.637	[0.397; 1.021]	0.061	0.718	[0.402; 1.282]	0.263
Duration of relationship Ref: < 3 years	> 3 years	3.001**	[1.761; 5.117]	< 0.001	0.356**	[0.202; 0.625]	< 0.001
Happiness with sexuality	Unhappy	0.262**	[0.153; 0.447]	< 0.001	0.252**	[0.111; 0.574]	0.001
Chronic pain	Yes	0.916	[0.507; 1.657]	0.773	1.488	[0.712; 3.111]	0.291
Intensity of pain Ref: 0–3	7–10	0.740	[0.359; 1.524]	0.414	0.726	[0.295; 1.790]	0.487
	4–6	0.583	[0.324; 1.049]	0.072	0.706	[0.338; 1.473]	0.353
Frequency of pain Ref: Yearly/Monthly	Daily	0.945	[0.509; 1.754]	0.857	0.655	[0.298; 1.436]	0.291
	Weekly	1.241	[0.635; 2.425]	0.528	0.584	[0.256; 1.334]	0.202
Dyspareunia	Yes	0.654**	[0.453; 0.943]	0.023	0.765	[0.491; 1.192]	0.236
Dysmenorrhea	Yes	0.854	[0.580; 1.259]	0.426	1.057	[0.662; 1.690]	0.815
Fatigue	Yes	0.564**	[0.379; 0.838]	0.005	0.721	[0.457; 1.137]	0.159
Infertility	Yes	0.658**	[0.448; 0.965]	0.032	1.008	[0.623; 1.631]	0.937

Dependent variable: total score of the PFB: 0: low/intermediate quality of partnership, 1: high quality of partnership.

** Statistically significant (*p* < 0.05).

PFB, Partnership questionnaire; OR, Odds ratio CI, Confidence interval.

**Table 4c:** Association between endometriosis as well as endometriosis related symptoms and happiness with the partnership in women and their partners.

		Women			Partners		
		OR	95% CI	*p* value	OR	95% CI	*p* value
Endometriosis	Yes	1.227	[0.570; 2.645]	0.601	1.103	[0.355; 3.425]	0.865
Number of children Ref: 0	> 2	1.006	[0.298; 3.400]	0.992	0.951	[0.180; 5.034]	0.953
	2	0.887	[0.368; 2.138]	0.789	0.154	[0.018; 1.284]	0.084
	1	0.910	[0.373; 2.223]	0.836	0.761	[0.218; 2.651]	0.761
Duration of relationship Ref: < 3 years	> 3 years	0.777	[0.308; 1.957]	0.592	0.601	[0.203; 1.776]	0.357
Happiness with sexuality	Unhappy	11.070**	[5.813; 21.084]	< 0.001	10.461**	[3.657; 29.927]	< 0.001
Chronic pain	Yes	0.850	[0.291; 2.483]	0.767	0.384	[0.074; 1.984]	0.253
Intensity of pain Ref: 0–3	7–10	0.869	[0.224; 3.381]	0.840	6.183**	[1.164; 32.849]	0.033
	4–6	1.344	[0.456; 3.960]	0.592	2.032	[0.376; 10.981]	0.410
Frequency of pain Ref: Yearly/Monthly	Daily	0.432	[0.131; 1.428]	0.169	1.007	[0.200; 5.071]	0.994
	Weekly	0.489	[0.147; 1.625]	0.243	1.131	[0.223; 5.727]	0.882
Dyspareunia	Yes	1.213	[0.602; 2.446]	0.589	0.916	[0.336; 2.494]	0.863
Dysmenorrhea	Yes	1.277	[0.626; 2.606]	0.502	0.874	[0.316; 2.421]	0.796
Fatigue	Yes	2.144	[0.958; 4.797]	0.063	1.114	[0.399; 3.113]	0.837
Infertility	Yes	1.318	[0.667; 2.605]	0.427	0.700	[0.242; 2.020]	0.509

Dependent variable: happiness with the relationship: 0: happy, 1: unhappy.

** Statistically significant (*p* < 0.05).

OR, Odds ratio; CI, Confidence interval.

**Table 4d:** Association between endometriosis as well as endometriosis related symptoms and the total score of the problem list (PL) in women and their partners.

		Women			Partners		
		OR	95% CI	*p* value	OR	95% CI	*p* value
Endometriosis	Yes	0.117	[0.709; 1.758]	0.634	0.950	[0.544; 1.661]	0.875
Number of children Ref: 0	> 2	1.392	[0.651; 2.979]	0.527	1.434	[0.587; 3.502]	0.429
	2	1.226	[0.725; 2.073]	0.447	1.025	[0.559; 1.880]	0.936
	1	1.183	[0.703; 1.991]	0.527	1.776	[0.901; 3.501]	0.097
Duration of relationship Ref: < 3 years	> 3 years	1.311	[0.796; 2.157]	0.287	0.569	[0.315; 1.127]	0.111
Happiness with sexuality	Unhappy	0.136**	[0.075; 0.246]	<0.001	0.266**	[0.131; 0.543]	< 0.001
Chronic pain	Yes	0.860**	[0.651; 0.947]	0.031	1.778	[0.776; 4.075]	0.174
Intensity of pain Ref: 0–3	7–10	0.426**	[0.199; 0.911]	0.028	0.676	[0.263;1.739]	0.417
	4–6	0.653	[0.343; 1.242]	0.194	0.844	[0.386; 1.845]	0.670
Frequency of pain Ref: Yearly/Monthly	Daily	0.767	[0.394; 1.492]	0.434	0.830	[0.352; 1.957]	0.670
	Weekly	0.792	[0.379; 1.656]	0.535	0.474	[0.201;1.116]	0.088
Dyspareunia	Yes	0.460**	[0.308; 0.688]	< 0.001	0.593**	[0.360; 0.976]	0.040
Dysmenorrhea	Yes	0.846	[0.561; 1.275]	0.424	0.811	[0.489; 1.346]	0.418
Fatigue	Yes	0.570**	[0.372; 0.874]	0.010	0.640	[0.383; 1.069]	0.088
Infertility	Yes	0.591**	[0.371; 0.727]	0.017	0.831	[0.783; 2.265]	0.291

Dependent variable: Total Score of the Problem list: 0: many conflict areas, 1: no/few conflict areas.

** Statistically significant (*p* < 0.05).

PL, Problem List; OR, Odds ratio; CI, Confidence interval.

Table 5 summarizes how often partners of women with endometriosis mention different feelings and strategies in the context of the disease. Correlation between these experiences and partner-ship quality are presented in Table 6. Women with endometriosis mentioned separation thoughts more often (34.9%) than control women (27.6%, *p* = 0.018). These thoughts were not significantly associated with endometriosis itself but with dissatisfaction with sexuality (OR: 2.212; CI: 1.307–3.743; *p* = 0.003) and with dyspareunia (OR: 1.566; CI: 1.070–2.292; *p* = 0.021). Fatigue (OR: 0.564; CI: 0.379–0.838; *p* = 0.005) and infertility (OR: 0.658; CI: 0.448–0.965; *p* = 0.032) were related to a lower assessment of the partnership quality. Women affected by endometriosis (2.00 ± 3.36) experienced more partnership-related conflicts than unaffected women (1.46 ± 2.91; *p* = 0.018). Chronic pain (OR: 0.947; CI: 651–0.947; *p* = 0.031), a higher intensity of pain (OR: 0.426; CI: 0.199–0.911; *p* = 0.028), dyspareunia (OR: 0.460; CI: 0.308–0.688; *p* < 0.001), dissatisfaction with the sexual relationship (OR: 0.426; CI: 0.199–0.911; *p* = 0.028), fatigue (OR: 0.570; CI: 0.372–0.874; *p* = 0.010), and infertility (OR: 0.591; CI: 0.371–0.727; *p* = 0.017) were significantly associated with unsolved conflicts and conflicts leading to severe disputes between women and their partners.

**Table 5:** Endometriosis-related experiences in 250 partners of women with endometriosis.

Parameter	Reported (very) often by partners of women with endometriosis
It is difficult for me to see my partner suffering	146 (58.4%)
I am worried about her health	172 (68.8%)
I am worried that we will never have children	53 (21.2%)
I am angry because endometriosis has such a strong impact on our life	47 (18.8%)
I think, if she pulled herself together, we could enjoy our life much more	11 (4.4%)
I am frustrated, that her symptoms do not get better	64 (25.6%)
I feel helpless in dealing with endometriosis	106 (42.4%)
I think that she exaggerates her suffering	7 (2.8%)
Endometriosis interferes with our sexual relationship	81 (32.4%)

**Table 6:** Correlation between different statements of male partners of women with endometriosis and partnership quality.

	r_s_	*p* value
It is difficult for me to see my partner suffering (1: rarely mentioned; 2: often mentioned)		
Happiness with relationship (1: happy; 2: unhappy)	0.121	0.057
Separation thoughts (1: yes; 2: no)	−0.026	0.680
Separation thoughts in connection with endometriosis (1: yes; 2: no)	0.028	0.706
Endometriosis as a burden in partnership (1: yes; 2: no)	−0.204	0.002*
I am worried about her health		
Happiness with relationship	0.162	0.011*
Separation thoughts	−0.107	0.093
Separation thoughts in connection with endometriosis	−0.025	0.737
Endometriosis as a burden in partnership	−0.135	0.039*
I am worried that we will never have children		
Happiness with relationship	−0.002	0.980
Separation thoughts	−0.052	0.413
Separation thoughts in connection with endometriosis	−0.098	0.193
Endometriosis as a burden in partnership	−0.091	0.165
I am angry because endometriosis has such a strong impact on our life		
Happiness with relationship	0.132	0.037*
Separation thoughts	−0.162	0.010*
Separation thoughts in connection with endometriosis	−0.207	0.006*
Endometriosis as a burden in partnership	−0.218	0.000*
I think, if she controlled herself better, we could enjoy our life much more		
Happiness with relationship	0.306	0.000*
Separation thoughts	−0.133	0.036*
Separation thoughts in connection with endometriosis	−0.265	0.000*
Endometriosis as a burden in partnership	−0.144	0.027*
I am frustrated that her symptoms do not get better		
Happiness with relationship	0.142	0.025*
Separation thoughts	−0.202	0.001*
Separation thoughts in connection with endometriosis	−0.158	0.036*
Endometriosis as a burden in partnership	−0.317	0.000*
I feel helpless in dealing with endometriosis		
Happiness with relationship	0.061	0.339
Separation thoughts	−0.167	0.008*
Separation thoughts in connection with endometriosis	−0.107	0.154
Endometriosis as a burden in partnership	−0.273	0.000*
I think that she exaggerates her suffering		
Happiness with relationship	0.134	0.034*
Separation thoughts	−0.094	0.138
Separation thoughts in connection with endometriosis	−0.159	0.034*
Endometriosis as a burden in partnership	−0.058	0.373
Endometriosis interferes with our sexual relationship		
Happiness with relationship	0.191	0.002*
Separation thoughts	−0.242	0.000*
Separation thoughts in connection with endometriosis	−0.352	0.000*
Endometriosis as a burden in partnership	−0.380	0.000*

Spearman Correlation Test; *Significant *p* < 0.05.

Male partners of women in both groups reported separation thoughts equally often (20.5%/25.2%; *p* = 0.131). In men, a high intensity of pain experienced by their female partner was associated with reduced partnership happiness (OR: 6.183; CI: 1.164–32.849; *p* = 0.033).

## Discussion

Women with endometriosis reported significantly more often thoughts about separation than control women (Table 3); this is in line with results in other chronic diseases (Buhmann et al., 2017; Karraker and Latham, 2015; Landfeldt et al., 2018). However, not endometriosis itself, but rather endometriosis-related factors such as a high intensity of pain, dyspareunia, or dissatisfaction with sexuality seem to play an important role in thoughts about separation (Table 4a). Interestingly, male partners of women with endometriosis did not reflect on separation more often than partners of control women. In couples living with endometriosis men reported even less often separation thoughts than their female partners. Instead, men seem to try to support their partners rather than considering leaving them; dealing with endometriosis may in fact bring partners closer together (Culley et al., 2013a,b; Denny, 2004; Culley et al., 2017; Fernandez et al., 2006; Hudson et al., 2016).

Consistent with findings from other studies, 40–50% of women and men affected by endometriosis perceive the disease as a strain on their partnership (Culley et al., 2017; Fourquet et al., 2010; Ameratunga et al., 2017). Notably, in 29% of cases where men contemplated ending their relationships, endometriosis was identified as the primary reason. Many partners struggled with witnessing their partners endure pain, expressed concern about her health, and experienced a sense of helplessness, all without undermining their partners’ coping abilities—a sentiment aligned with earlier research findings (Culley et al., 2013a; Denny, 2004; Culley et al., 2017; Ameratunga et al., 2017). As expected, chronic pain and fatigue were often mentioned as the reason for suffering and reduced capability (Ramin-Wright et al., 2018; Butt and Chesla, 2007; De Graaff et al., 2016). Feelings such as anger, frustration, and thoughts that women exaggerate or should control themselves have a significant negative impact on relationship quality (Fernandez et al., 2006). In contrast to other studies, age was not related to thoughts about separation (Karraker and Latham, 2015; Landfeldt et al., 2018).

As to be expected, separation thoughts were associated with reduced partnership happiness. Overall partnership quality and partnership happiness were not significantly different between women with and without endometriosis and between the two groups of male partners. Generally, women rate the quality of a partnership higher than men (Hahlweg, 2016; Müller et al., 2001), which is also confirmed in our study. Only male partners of women with endometriosis showed a correlation between a high intensity of pain in their partners and unhappiness in the partnership. These findings are in contrast to those in chronic diseases such as multiple sclerosis, Huntington’s disease, erectile dysfunction, etc. (Buhmann et al., 2017; Müller et al., 2001; Rosen, 2001; Baanders and Heijmans, 2007; Reininghaus et al., 2012), i.e., couples facing endometriosis seem to succeed particularly well in resolving partnership-related challenges. Individual variances in frequency and intensity of disease symptoms might also add to the differences in findings. In women with endometriosis and in control women, a long-term relationship was associated with a better overall partnership quality as well as with higher scores in “tenderness,” “togetherness,” and “communication,” while men showed the opposite results. Consequently, changes associated with the duration of a partnership (including when dealing with endometriosis) seem to be experienced differently by men and women; this should be considered in counseling. As women with a more recent diagnosis of endometriosis reported to think particularly often about separation, reflections on the impact of the disease on partnership, seem to be an important aspect of adapting to the disease.

Our findings (Tables 3, 4c) confirm that sexual satisfaction is an important factor in relationship happiness, also in the context of endometriosis (Culley et al., 2013a,b; Culley et al., 2017; Hämmerli et al., 2018; Ameratunga et al., 2017; Schick et al., 2022; Reininghaus et al., 2012). In women with endometriosis, dyspareunia showed a correlation with a lower overall partnership quality. Although fatigue was not directly associated with lower partnership happiness or separation thoughts, it might add to sexual problems (Ramin-Wright et al., 2018; De Graaff et al., 2016). Other studies reported no association between sexual problems (e.g., erectile dysfunction) and partnership quality (Müller et al., 2001); this may depend on whether the male or the female partner is affected. Also, which sexual activities are investigated might play a role. Most studies reporting lower partnership happiness in association with endometriosis concentrated on sexuality, especially on the frequency of sexual intercourse (Culley et al., 2013a,b; Culley et al., 2017; Ameratunga et al., 2017). In line with other authors our results show a similar frequency of caresses and kissing in couples with endometriosis and those without (Hämmerli et al., 2018; Ameratunga et al., 2017). Men stated more frequently that they were only rarely caressed by their partners; this concern contrasts with the assumption that men prefer a coitus-centered romantic relationship (Hämmerli et al., 2018).

Although pain is strongly related to partnership quality in the context of endometriosis, other factors and disease symptoms influence this association. For example, the number of children seems to be relevant for partnership happiness. General relationship happiness was lower in couples with a higher number of children, whether affected by endometriosis or not; this is probably due to a focus on childcare instead of on the partnership. Also, infertility was associated with lower partnership quality, which is probably due to increased conflicts, lacking or inadequate communication, and isolation (Andrews et al., 1991; Hadley and Hanley, 2011; Kiesswetter et al., 2020; Lowyck et al., 2009; Wischmann et al., 2001). Although endometriosis-related infertility seems to be a source of conflict between women and their partners (Butt and Chesla, 2007; Hudson et al., 2016; Hadley and Hanley, 2011; Lowyck et al., 2009; Wischmann et al., 2001; Dunselman et al., 2014), infertility was not related to consideration of separation, i.e., most couples succeeded in preventing break-up of the partnership because of infertility and find other sources of satisfaction (Andrews et al., 1991; Kiesswetter et al., 2020). Fatigue was associated with reduced partnership quality and dealing with tenderness. Fatigue might be a reason why women are not in the mood for sexual activity, but less interest in sexual activity is also associated with stress, depression, and chronic pain, all of which might indirectly affect tenderness (Ramin-Wright et al., 2018).

In agreement with previous research women with endometriosis reported more frequent conflicts and unsolved problems than control women (Martire et al., 2019; Rosland et al., 2012). Women with endometriosis reported more often problems with self-confidence, feelings of attractivity, femininity, trust, and jealousness, as well as their consequences on social and sexual encounters, than control women. On this basis, sexuality represents a source of conflicts (Hämmerli et al., 2018; Fourquet et al., 2010; Ameratunga et al., 2017; De Graaff et al., 2016; Fagervold et al., 2009). In contrast to control women, women with endometriosis see their partners’ temperament as a conflict area; this might be explained by feelings of frustration and anger in reaction to endometriosis-related symptoms, especially chronic pain and a high intensity of pain (Tables 4d, 5) (Culley et al., 2017; Fernandez et al., 2006; De Graaff et al., 2016). Fatigue also is associated with a higher number of unsolved conflicts in women with endometriosis. Fatigue and related factors such as depression, insomnia, and pain might influence argumentative behavior (Ramin-Wright et al., 2018). Male partners of women with endometriosis reported conflicts about household issues, probably due to the higher demands resulting from the health restrictions of their partners (Culley et al., 2017). Interestingly, endometriosis-affected women reported fewer conflicts regarding lack of support and of acceptance by their partners than control women; which may also be related to the low rates of separation thoughts and similar prevalences of partnership happiness in both groups. Irrespective of the presence of endometriosis, male partners report more often that they are blamed by their partners and that they have to face endless conflicts; this could be a result of different partnership communication strategies in men and women (Greeff and De Bruyne, 2000). Altogether, the PL represents a valuable tool to identify conflict areas and resources to improve counseling in women and couples facing endometriosis.

Given that partnership quality serves as a crucial resource in managing endometriosis, encompassing symptoms such as pain, and enhancing overall quality of life (Schick et al., 2022), it is imperative that medical counseling for women diagnosed with endometriosis and their partners includes support mechanisms to effectively address the impact of endometriosis on their partnership. In addition to educating the partner about disease symptoms, both couple and sexual counseling may help to increase well-being in couples affected by chronic diseases such as endometriosis (Golics et al., 2013; Hadley and Hanley, 2011; Hartmann et al., 2010; Lowyck et al., 2009; Martire et al., 2019; Rosland et al., 2012; Schick et al., 2022; Wischmann et al., 2001).

### Strengths and limitations

The three greatest strengths of this study are the large study group collected in different German-speaking countries, the comparison of women with endometriosis and matched control women, and the inclusion of both partners. In addition, we present one of the first investigations focusing on the quality of partnership and not on sexuality only.

Recruitment in the context of gynecological consultations and procedures allowed an unselected sample with regard to partnership problems. A high percentage of women who completed the questionnaire were in partnerships at the time; however, women with a very low partnership quality might have refrained from completing this part of the questionnaire. Since of the 467 women diagnosed with endometriosis and 458 control women who were in a partnership, only 28 women with endometriosis and 21 women in the control group did not complete the PFB, we do not think such selection bias has relevantly influenced our findings. Male partners were recruited through their female partners. Therefore, men who were in well-functioning partnerships might be overrepresented in our sample. In both women and their partners, possible selection bias would result in underestimation of our findings.

The number of returned partner questionnaires was lower than the number of completed women’s questionnaires, but with over 60% of returned partner questionnaires in both groups, return rate of male responses is very satisfactory. However, as the smaller number of partners resulted in a reduction in power these results should be interpreted with caution. Diseases or symptoms in men that could have an effect on the quality of partnership were not evaluated. As only heterosexual partners were included, we cannot provide any information on female partners.

Despite the exclusion of women with potential endometriosis-related symptoms, the control group might include women with an asymptomatic undiagnosed endometriosis. As endometriosis-related symptoms have been shown to be the main factor in the association between endometriosis and partnership quality, such misclassification should have no significant impact on our findings.

The cross-sectional design of the study does not allow any conclusions on causality.

## Conclusion

Symptoms linked to endometriosis, such as chronic pain and dyspareunia, are correlated with diminished partnership satisfaction and thoughts of separation, while fatigue appears to have a lesser impact. This chronic gynecological pain disorder is associated with a heightened prevalence of unresolved conflicts between women and their partners, particularly in the realms of sexuality and infertility. Considering the pivotal role partnership plays in coping with this disabling condition and its overall impact on quality of life, medical counseling should involve both partners. Addressing partnership issues and implementing strategies to alleviate the burdens posed by endometriosis on partnership should be an integral component of medical counseling.

## Data Availability

The raw data of the study will be distributed upon reasonable request.
