# Supplementary material for: Partnership and relationship happiness in endometriosis related chronic pelvic pain: a multicenter case–control study
Source: Front Psychol. 2024 Oct 14;15:1382067. doi: 10.3389/fpsyg.2024.1382067 (PMC11514139; doi:10.3389/fpsyg.2024.1382067)
Supplement: Supplementary file 1 [file Table_1.docx]

Supplementary Table 4e: Association between endometriosis as well as endometriosis related symptoms and the sub-score argumentative behavior in women and their partners

|  |  |  | Women |  |  | Partners |  |  |
| --- | --- | --- | --- | --- | --- | --- | --- | --- |
|  |  |  | OR | 95% CI | *P* value | OR | 95% CI | *P* value |
| Endometriosis | Yes |  | 1.078 | [0.362;3.212] | 0.892 | 0.663 | [0.246;1.785] | 0.416 |
|  | No | Ref. |  |  |  |  |  |  |
| Number of children | > 2 |  | 1.314 | [0.156;11.065] | 0.802 | 1.956 | [0.239;15.982] | 0.531 |
|  | 2 |  | 0.678 | [0.213;2.156] | 0.510 | 0.681 | [0.256;1.813] | 0.442 |
|  | 1 |  | 0.761 | [0.232;2.499] | 0.652 | 1.150 | [0.359;3.687] | 0.814 |
|  | 0 | Ref. |  |  |  |  |  |  |
| Duration of relationship | > 3 years |  | 0.951 | [0.293;3.087] | 0.934 | 0.553 | [0.152;2.013] | 0.368 |
|  | < 3 years | Ref. |  |  |  |  |  |  |
| Happiness with sexuality | Unhappy |  | 0.479 | [0.164;1.393] | 0.176 | 0.643 | [0.221;1.868] | 0.417 |
|  | Happy | Ref. |  |  |  |  |  |  |
| Chronic pain | Yes |  | 1.273 | [0.236;6.851] | 0.779 | 1.227 | [0.318;4.725] | 0.767 |
|  | No | Ref. |  |  |  |  |  |  |
| Frequency of pain | Daily |  | 1.691 | [0.452;6.324] | 0.435 | 0.836 | [0.207;3.370] | 0.801 |
|  | Weekly |  | 2.365 | [0.454;12.316] | 0.307 | 0.516 | [0.145;1.838] | 0.307 |
|  | Yearly/Monthly | Ref. |  |  |  |  |  |  |
| Intensity of pain | 7-10 |  | 0.214 | [0.036;1.288] | 0.092 | 0.534 | [0.131;2.173] | 0.381 |
|  | 4-6 |  | 0.205 | [0.039;1.071] | 0.060 | 0.764 | [0.225;2.587] | 0.665 |
|  | 0-3 | Ref. |  |  |  |  |  |  |
| Dyspareunia | Yes |  | 0.628 | [0.231;1.709] | 0.362 | 0.711 | [0.291;1.740] | 0.456 |
|  | No | Ref. |  |  |  |  |  |  |
| Dysmenorrhea | Yes |  | 1.000 | [0.357;2.803] | 1.000 | 1,213 | [0.478;3.075] | 0.685 |
|  | No | Ref. |  |  |  |  |  |  |
| Fatigue | Yes |  | 0.632 | [0.195;2.048] | 0.444 | 0.541 | [0.190;1.538] | 0.249 |
|  | No | Ref. |  |  |  |  |  |  |
| Infertility | Yes |  | 1.027 | [0.381;2.768] | 0.958 | 0.850 | [0.353;2.049] | 0.717 |
|  | No | Ref |  |  |  |  |  |  |

*Notes.* Dependent variable: argumentative behavior: 0 for low/intermediate quality of partnership, 1 for high quality of partnership were used.

** Statistically significant (p < 0,05).

OR = Odds ratio CI =Confidence interval

Supplementary Table 4f: Association between endometriosis as well as endometriosis related symptoms and the sub-score tenderness in women and their partners

|  |  |  | Women |  |  | Partners |  |  |
| --- | --- | --- | --- | --- | --- | --- | --- | --- |
|  |  |  | OR | 95% CI | *P* value | OR | 95% CI | *P* value |
| Endometriosis | Yes |  | 0.908 | [0.578;1.427] | 0.677 | 0.856 | [0.523;1.399] | 0.534 |
|  | No | Ref. |  |  |  |  |  |  |
| Number of children | > 2 |  | 0.413** | [0.212;0.801] | 0.009 | 0.359** | [0.169;0.761] | 0.008 |
|  | 2 |  | 0.374** | [0.230;0.610] | < 0.001 | 0.418** | [0.243;0.720] | 0.002 |
|  | 1 |  | 0.574** | [0.338;0.976] | 0.040 | 0.600 | [0.335;1.075] | 0.086 |
|  | 0 | Ref. |  |  |  |  |  |  |
| Duration of relationship | > 3 years |  | 3.810** | [1.831;7.931] | < 0.001 | 0.398** | [0.212;0.749] | 0.004 |
|  | < 3 years | Ref. |  |  |  |  |  |  |
| Happiness with sexuality | Unhappy |  | 0.182** | [0.107;0.311] | < 0.001 | 0.246** | [0.119;0.509] | < 0.001 |
|  | Happy | Ref. |  |  |  |  |  |  |
| Chronic pain | Yes |  | 0.846 | [0.434;1.651] | 0.625 | 1.384 | [0.653;2.930] | 0.397 |
|  | No | Ref. |  |  |  |  |  |  |
| Intensity of pain | 7-10 |  | 1.426 | [0.599;3.394] | 0.422 | 1.215 | [0.478;3.091] | 0.683 |
|  | 4-6 |  | 0.569 | [0.300;1.079] | 0.084 | 0.632 | [0.304;1.314] | 0.219 |
|  | 0-3 | Ref. |  |  |  |  |  |  |
| Frequency of pain | Daily |  | 0.931 | [0.463;1.874] | 0.842 | 0.787 | [0.351;1.764] | 0.561 |
|  | Weekly |  | 0.912 | [0.439;1.893] | 0.805 | 0.615 | [0.273;1.386] | 0.241 |
|  | Yearly/Monthly | Ref. |  |  |  |  |  |  |
| Dyspareunia | Yes |  | 0.847 | [0.558;1.286] | 0.437 | 0.786 | [0.498;1.242] | 0.302 |
|  | No | Ref. |  |  |  |  |  |  |
| Dysmenorrhea | Yes |  | 0.995 | [0.641;1.542] | 0.980 | 1.182 | [0.733;1.907] | 0.494 |
|  | No | Ref. |  |  |  |  |  |  |
| Fatigue | Yes |  | 0.620** | [0.394;0.974] | 0.038 | 0.983 | [0.618;1.565] | 0.944 |
|  | No | Ref. |  |  |  |  |  |  |
| Infertility | Yes |  | 0.600** | [0.393;0.916] | 0.018 | 0.961 | [0.592;1.562] | 0.874 |
|  | No | Ref |  |  |  |  |  |  |

*Notes.* Dependent variable: tenderness: 0 for low/intermediate quality of partnership, 1 for high quality of partnership were used.

** Statistically significant (*P* < 0,05).

OR = Odds ratio CI =Confidence interval

Supplementary Table 4g: Association between endometriosis as well as endometriosis related symptoms and the sub-score togetherness and communication in women and their partners

|  |  |  | Women |  |  | Partners |  |  |
| --- | --- | --- | --- | --- | --- | --- | --- | --- |
|  |  |  | OR | 95% CI | *P* value | OR | 95% CI | *P* value |
| Endometriosis | Yes |  | 0.896 | [0.587;1.368] | 0.611 | 1.070 | [0.622;1.841] | 0.806 |
|  | No | Ref. |  |  |  |  |  |  |
| Number of children | > 2 |  | 0.463** | [0.244;0.878] | 0.018 | 0.255** | [0.119;0.547] | < 0.001 |
|  | 2 |  | 0.481** | [0.302;0.768] | 0.002 | 0.334** | [0.189;0.590] | < 0.001 |
|  | 1 |  | 0.506** | [0.309;0.826] | 0.006 | 0.552 | [0.293;1.038] | 0.065 |
|  | 0 | Ref. |  |  |  |  |  |  |
| Duration of relationship | > 3 years |  | 2.939** | [1.598;5.406] | 0.001 | 0.428** | [0.198;0.925] | 0.031 |
|  | < 3 years | Ref. |  |  |  |  |  |  |
| Happiness with sexuality | Unhappy |  | 0.262** | [0.156;0.440] | < 0.001 | 0.408** | [0.207;0.802] | 0.009 |
|  | Happy | Ref. |  |  |  |  |  |  |
| Chronic pain | Yes |  | 0.859 | [0.458;1.611] | 0.636 | 0.888 | [0.394;1.998] | 0.774 |
|  | No | Ref. |  |  |  |  |  |  |
| Intensity of pain | 7-10 |  | 1.428 | [0.655;3.113] | 0.370 | 1.466 | [0.496;4.331] | 0.489 |
|  | 4-6 |  | 1.176 | [0.638;2.168] | 0.603 | 0.736 | [0.335;1.613] | 0.444 |
|  | 0-3 | Ref. |  |  |  |  |  |  |
| Frequency of pain | Daily |  | 0.546 | [0.285;1.046] | 0.068 | 1.267 | [0.505;3.178] | 0.614 |
|  | Weekly |  | 0.930 | [0.460;1.880] | 0.839 | 0.758 | [0.323;1.780] | 0.524 |
|  | Yearly/Monthly | Ref. |  |  |  |  |  |  |
| Dyspareunia | Yes |  | 0.661** | [0.448;0.975] | 0.037 | 1.008 | [0.607;1.672] | 0.975 |
|  | No | Ref. |  |  |  |  |  |  |
| Dysmenorrhea | Yes |  | 0.992 | [0.659;1.494] | 0.970 | 0.881 | [0.521;1.489] | 0.636 |
|  | No | Ref. |  |  |  |  |  |  |
| Fatigue | Yes |  | 0.751 | [0.494;1.414] | 0.180 | 1.004 | [0.605;1.667] | 0.987 |
|  | No | Ref. |  |  |  |  |  |  |
| Infertility | Yes |  | 0.460** | [0.307;0.689] | < 0.001 | 1.060 | [0.622;1. 806] | 0.830 |
|  | No | Ref |  |  |  |  |  |  |

*Notes.* Dependent variable: total score of the PFB: 0 for low/intermediate quality of partnership, 1 for high quality of partnership were used.

** Statistically significant (p < 0,05).

OR = Odds ratio CI =Confidence interval

Supplementary Table 4h: Association between endometriosis as well as endometriosis related symptoms and the sub-score burden in the partnership in women and their partners

|  |  |  | Women |  |  | Partners |  |  |
| --- | --- | --- | --- | --- | --- | --- | --- | --- |
|  |  |  | OR | 95% CI | *P* value | OR | 95% CI | *P* value |
| Endometriosis | Yes |  | 1.426 | [0.408;4.979] | 0.578 | 0.580 | [0.206;1.636] | 0.303 |
|  | No | Ref. |  |  |  |  |  |  |
| Number of children | > 2 |  | 0.345 | [0.087;1.367] | 0.130 | 2.816 | [0.662;11.991] | 0.161 |
|  | 2 |  | 1.368 | [0.625;2.993] | 0.433 | 1.533 | [0.640;3.669] | 0.338 |
|  | 1 |  | 0.835 | [0.446;1.918] | 0.835 | 1.482 | [0.625;3.515] | 0.372 |
|  | 0 | Ref. |  |  |  |  |  |  |
| Duration of relationship | > 3 years |  | 0.676 | [0.339;1.348] | 0.267 | 0.642 | [0.271;1.519] | 0.313 |
|  | < 3 years | Ref. |  |  |  |  |  |  |
| Happiness with sexuality | Unhappy |  | 1.733 | [0.841;3.574] | 0.136 | 1.305 | [0.522;3.258] | 0.569 |
|  | Happy | Ref. |  |  |  |  |  |  |
| Chronic pain | Yes |  | 1.651 | [0.823;3.312] | 0.158 | 0.605 | [0.231;1.587] | 0.307 |
|  | No | Ref. |  |  |  |  |  |  |
| Intensity of pain | 7-10 |  | 1.263 | [0.577;2.764] | 0.559 | 2.638 | [0.957;7.277] | 0.061 |
|  | 4-6 |  | 2.337** | [1.186;4.605] | 0.014 | 4.147 | [1.688;10.187] | 0.638 |
|  | 0-3 | Ref. |  |  |  |  |  |  |
| Frequency of pain | Daily |  | 2.169** | [1.048;4.487] | 0.037 | 1.501 | [0.594;3.793] | 0.390 |
|  | Weekly |  | 0.960 | [0.458;2.012] | 0.914 | 1.461 | [0.575;3.715] | 0.426 |
|  | Yearly/Monthly | Ref. |  |  |  |  |  |  |
| Dyspareunia | Yes |  | 1.445 | [0.837;2.495] | 0.187 | 1.962 | [0.968;3.977] | 0.061 |
|  | No | Ref. |  |  |  |  |  |  |
| Dysmenorrhea | Yes |  | 1.118** | [0.638;0.959] | 0.001 | 0.854 | [0.424;1.719] | 0.658 |
|  |  |  |  |  |  |  |  |  |
| Fatigue | Yes |  | 2.616** | [1.337;5.118] | 0.005 | 0.772 | [0.358;1.666] | 0.509 |
|  | No | Ref. |  |  |  |  |  |  |
| Infertility | Yes |  | 1.013 | [0.607;1.692] | 0.961 | 1.278 | [0.664;2.460] | 0.463 |
|  | No | Ref |  |  |  |  |  |  |

*Notes.* Dependent variable: burden in partnership: 0 for no, 1 for yes were used.

** Statistically significant (*P* < 0,05).

OR = Odds ratio CI =Confidence interval
